# Systematic polypharmacology and drug repurposing via an integrated L1000-based Connectivity Map database mining

**DOI:** 10.1098/rsos.181321

**Published:** 2018-11-28

**Authors:** Tsang-Pai Liu, Yao-Yu Hsieh, Chia-Jung Chou, Pei-Ming Yang

**Affiliations:** 1 PhD Program for Cancer Molecular Biology and Drug Discovery, College of Medical Science and Technology, Taipei Medical University and Academia Sinica, Taipei, Taiwan, Republic of China; 2 Department of Surgery, Mackay Memorial Hospital, Taipei, Taiwan, Republic of China; 3 Liver Medical Center, Mackay Memorial Hospital, Taipei, Taiwan, Republic of China; 4 Mackay Junior College of Medicine, Nursing and Management, New Taipei City, Taiwan, Republic of China; 5 Department of Medicine, Mackay Medical College, New Taipei City, Taiwan, Republic of China; 6 Division of Hematology and Oncology, Taipei Medical University Shuang Ho Hospital, New Taipei City, Taiwan, Republic of China; 7 Division of Hematology and Oncology, Department of Internal Medicine, School of Medicine, College of Medicine, Taipei Medical University, Taipei, Taiwan, Republic of China; 8 Graduate Institute of Cancer Biology and Drug Discovery, College of Medical Science and Technology, Taipei Medical University, Taipei, Taiwan, Republic of China; 9 TMU Research Center of Cancer Translational Medicine, Taipei, Taiwan, Republic of China

**Keywords:** cancer therapy, Connectivity Map, drug repurposing, histone deacetylase, polypharmacology, topoisomerase

## Abstract

Drug repurposing aims to find novel indications of clinically used or experimental drugs. Because drug data already exist, drug repurposing may save time and cost, and bypass safety concerns. Polypharmacology, one drug with multiple targets, serves as a basis for drug repurposing. Large-scale databases have been accumulated in recent years, and utilization and integration of these databases would be highly helpful for polypharmacology and drug repurposing. The Connectivity Map (CMap) is a database collecting gene-expression profiles of drug-treated human cancer cells, which has been widely used for investigation of polypharmacology and drug repurposing. In this study, we integrated the next-generation L1000-based CMap and an analytic Web tool, the L1000FWD, for systematic analyses of polypharmacology and drug repurposing. Two different types of anti-cancer drugs were used as proof-of-concept examples, including histone deacetylase (HDAC) inhibitors and topoisomerase inhibitors. We identified KM-00927 and BRD-K75081836 as novel HDAC inhibitors and mitomycin C as a topoisomerase IIB inhibitor. Our study provides a prime example of utilization and integration of the freely available public resources for systematic polypharmacology analysis and drug repurposing.

## 1. Introduction

Drug repurposing, also known as drug repositioning, aims to identify novel indication(s) of an approved or experimental drug [1]. Polypharmacology describes the ability of a drug to affect more than one molecular target, which has been viewed as a basic property of many therapeutic small molecules and serves as a principle for drug repurposing [2]. Because the safety and efficacy of an approved drug has already been optimized for its original indication, the time for a new indication approval may be significantly shorter and the cost is likely to be far less than those for a new drug. The Connectivity Map (CMap) is a database that collects microarray-based gene-expression profiles from cultured human cancer cell lines treated with various experimentally and clinically used small molecules, and provides a pattern-matching Web-based software to mine these data [3,4]. The current version (build 02; https://portals.broadinstitute.org/cmap/) of CMap collects more than 7000 gene-expression profiles representing 1309 compounds. Because most CMap compounds are the United States Food and Drug Administration (FDA)-approved drugs, this database has become a powerful tool for drug repurposing. By inputting a gene-expression profile of interest and querying it against the CMap data, a list of ranked CMap drugs is obtained. Drugs with positive scores indicate that these CMap drugs and the queried drug have similar gene-expressing signatures, and may share similar mechanism-of-action (MOA). Thus, the queried drug may be able to treat the same diseases as the original indications of these CMap drugs. Moreover, CMap drugs can be repurposed for novel indications if the gene-expression signatures of established clinical drugs are queried.

Conventional microarray-based gene-expression profiling is expensive and not suitable for high-throughput small-molecule screening. The L1000 assay platform is a new low-cost and high-throughput gene-expression profiling method, which contains 1000 landmark transcripts used to estimate the genome-wide gene-expression signature generated by microarrays [5,6]. L1000 assay combines ligation-mediated amplification, optically addressed and barcoded microspheres (beads), and flow cytometric detection system for gene-expression signature analysis [6]. Based on L1000 assay platform, the Library of Integrated Cellular Signatures (LINCS) is then developed as the next-generation version of CMap [5,7]. Compared to CMap, the LINCS collects the gene-expression profiles of not only small-molecule treatments but also genetic manipulation for knocking-down genes by shRNA or over-expressing genes by cDNA. In addition, the LINCS has higher numbers of gene-expression signatures (8870 perturbagens) and cell lines (nine cancer cell lines). Previously, LINCS was accessed via a Web-based interface, the Lincscloud (http://www.lincscloud.org/). Recently, the Lincscloud has been deprecated and replaced by the CLUE (https://clue.io/).

There are several useful resources and databases that are derived from L1000-based LINCS data. For example, the L1000 Characteristic Direction Signature Search Engine (L1000CDS2; http://amp.pharm.mssm.edu/L1000CDS2/) is designed to query gene-expression signatures against the LINCS data to discover and prioritize small molecules that reverse or mimic the inputted gene-expression profile [8]. The CRowd Extracted Expression of Differential Signatures (CREEDS; http://amp.pharm.mssm.edu/CREEDS/) combines gene-expression profiles from the gene-expression omnibus (GEO), a repository for omics data [9], with LINCS data to identify potential novel drug mimickers [10]. The L1000 Fireworks Display (L1000FWD; http://amp.pharm.mssm.edu/L1000FWD/) provides visualization of drug-induced gene-expression signatures and their similarity in MOA [11]. The integrative LINCS (iLINCS; http://www.ilincs.org/ilincs/) is an integrative Web platform for analysing transcriptomics and proteomics LINCS data. Given the fact that increasingly large-scale databases and analytic tools are developed, the strategies of utilization and integration of these resources have become more and more important.

In this study, we attempt to establish a systematic analysis strategy for polypharmacology and drug repurposing by the integration of the next-generation CMap and L1000FWD. Two types of anti-cancer drugs were used as proof-of-concept examples, including histone deacetylase (HDAC) inhibitors and topoisomerase inhibitors. Our results indicated that the L1000-based next-generation CMap had a convenient and reliable prediction for drug repurposing and polypharmacology. Accompanied analysis of candidate drugs by the L1000FWD further excluded the drugs with dissimilar gene signatures. Two novel HDAC inhibitors (KM-00927 and BRD-K75081836) and a novel topoisomerase IIB inhibitor (mitomycin C) were identified. Taken together, this study gives a prime example of systematic polypharmacology analysis and drug repurposing by integrating the freely available resources.

## 2. Results and discussion

### 2.1. Prediction of novel histone deacetylase inhibitors by the Connectivity Map

Histone acetylation by the histone acetyltransferases (HATs) and deacetylation by the HDACs is essential for regulating gene transcription in eukaryotic cells [12]. The addition of acetyl groups to lysine residues of histones promotes a more-relaxed chromatin structure, allowing transcriptional activation. By contrast, the removal of acetyl group makes the chromatin structure more compact, resulting in gene silencing [13,14]. Because over-expression of HDACs was frequently found in tumours, which prevents tumour suppressor gene expressions, inhibition of HDACs is considered a potential anti-cancer strategy. Currently, several HDAC inhibitors have been approved by the FDA for clinical uses. Vorinostat (suberoylanilide hydroxamic acid; SAHA) and romidepsin (depsipeptide or FK228), have been approved for treating relapsed cutaneous T-cell lymphoma in 2006 and 2009, respectively [15,16]. Panobinostat has been approved for treating multiple myeloma in 2015 [17]. Belinostat has been approved for treating peripheral T-cell lymphoma in 2014 [18].

**Figure 1.**
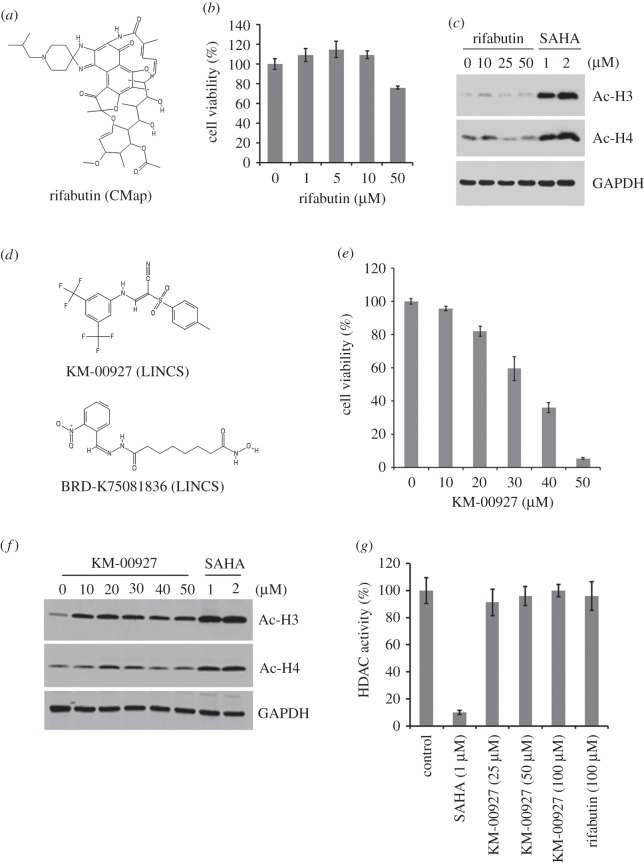
Effect of predicted HDAC inhibitors on cell viability and histone acetylation. (*a*) The chemical structure of rifabutin. (*b*) HCT116 cells were treated with various doses of rifabutin for 72 h, and then cell viability was measured with an MTT assay. (*c*) HCT116 cells were treated with various doses of rifabutin or SAHA for 24 h, and protein expressions of acetyl-histones H3 and H4, and GAPDH were analysed by Western blotting. (*d*) The chemical structures of KM-00927 and BRD-K75081836. (*e*) HCT116 cells were treated with various doses of KM-00927 for 72 h, and then cell viability was measured with an MTT assay. (*f*) HCT116 cells were treated with various doses of KM-00927 or SAHA for 24 h, and protein expressions of acetyl-histones H3 and H4, and GAPDH were analysed by Western blotting. (*g*) Nuclear protein lysates were incubated with various doses of KM-00927, 100 µM rifabutin or 5 µM SAHA in HDAC assay buffer for 1 h. HDAC activity was initiated by adding the HDAC substrate and incubating at 37°C for 1 h. HDAC activity was measured by detecting the OD value at 405 nm.

Transcriptional reprogramming is believed to contribute largely to the therapeutic benefits of HDAC inhibitors [19]. Because CMap is established based on drug transcriptome profiles, we proposed that CMap is a suitable tool for the discovery of novel HDAC inhibitors by comparing the similarity of gene-expression profiling (gene signatures) between the established HDAC inhibitors and CMap drugs. Differentially expressed genes (DEGs, listed in electronic supplementary material, table S1) in HDAC inhibitor-treated cancer cells, including SAHA-treated HCT116 (GSE22061 [20]), SAHA-treated MDA-MB-231 (GSE60125) and FK228-treated HCT116 cells (GSE22061 [20]), were prepared using the GEO online tool GEO2R [9]. These DEGs were queried using CMap (build 02; https://portals.broadinstitute.org/cmap/), and the results were shown in table 1 ranked according to the average scores. CMap drugs with positive mean scores share similar gene-expression patterns with the queried HDAC inhibitors. We found that several HDAC inhibitors (SAHA/vorinostat, trichostatin A, scriptaid, HC toxin and MS-275) were similar to rifabutin (table 1; its chemical structure as shown in figure 1*a*). Rifabutin is an anti-mycobacterial agent primarily used in the treatment of tuberculosis. Because rifabutin is a semisynthetic ansamycin that has been found to be a heat shock protein 90 (HSP90) inhibitor [21], the mean scores of common HSP90 inhibitors (geldanamycin, tanespimycin, alvespimycin) were also shown as a comparison (table 1). However, these HSP90 inhibitors were not similar to HDAC inhibitors.

**Table 1.** Prediction of potential HDAC inhibitors by the CMap. Differentially expressed genes (DEGs) induced by HDAC inhibitors were obtained from NCBI GEO database, including GSE22061 (SAHA- and FK228-treated HCT116 cells) and GSE60125 (SAHA-treated MDA-MB-231 cells). The full list of DEGs is shown in electronic supplementary material, table S1. These DEGs were inputted to the CMap build 02 and similar drugs were obtained. The mean scores of common HDAC and HSP90 inhibitors are listed in this table, which is ranked by average score that is calculated by adding up the mean scores and dividing the total by the numbers of scores.

			queried microarray datasets					
			HCT116-SAHA		HCT116-FK228		MDA-MB-231-SAHA	
CMap drug	function	average score	mean score	*p*-value	mean score	*p*-value	mean score	*p*-value
Scriptaid	HDAC inhibitor	0.807	0.831	0.00004	0.812	0.00004	0.777	0.00004
HC toxin	HDAC inhibitor	0.803	0.853	—	0.823	—	0.733	—
SAHA	HDAC inhibitor	0.801	0.831	0	0.827	0	0.746	0
Trichostatin A	HDAC inhibitor	0.716	0.721	0	0.703	0	0.723	0
Rifabutin	anti-mycobacterial agent; HSP90 inhibitor	0.652	0.651	0.00004	0.541	0.00004	0.765	0.00004
MS-275	class I HDAC inhibitor	0.327	0.191	0.16213	0	—	0.791	0.00016
Geldanamycin	HSP90 inhibitor	0.173	0.175	0	0.116	0.03101	0.229	0.06665
Tanespimycin	HSP90 inhibitor	0.172	0.159	0	0.089	0	0.268	0
Alvespimycin	HSP90 inhibitor	0.155	0.131	0.02932	0.044	0.01495	0.291	0.00008
Monorden	HSP90 inhibitor	0.08	0.111	0.00092	0	—	0.128	0.07013

To determine the anti-cancer activity of rifabutin against HCT116 cells, MTT assay was performed. The doses of rifabutin less than 10 µM slightly increased the cell viability whereas 50 µM rifabutin reduced the cell viability to 76% (figure 1*b*). Inhibition of HDAC activity can induce the acetylation of histones. To examine the effect of rifabutin on HDAC activity, whole cell lysates from HCT116 cells treated with rifabutin and SAHA (a positive control) were analysed by Western blotting. As shown in figure 1*c*, the expression of acetylated histones H3 and H4 increased in response to SAHA, but not rifabutin treatment. Therefore, rifabutin is not an HDAC inhibitor.

### 2.2. Prediction of novel histone deacetylase inhibitors by the Library of Integrated Cellular Signatures

Because the next-generation CMap, the LINCS, has higher numbers of drug-gene signatures than CMap, we also used the LINCS to predict potential HDAC inhibitors. By querying LINCS data with the same DEGs as described above (electronic supplementary material, table S1), we found that two drugs (KM-00927 and BRD-K75081836; their chemical structures are shown in figure 1*d*) were similar to HDAC inhibitors (table 2). Because only KM-00927 is commercially available, its effects on cell viability and histone H3/H4 acetylation were examined. As shown in figure 1*e*, KM-00927 dose-dependently killed HCT116 cells. In addition, the acetylation of histones H3/H4 was induced by KM-00927, as SAHA did (figure 1*f*), suggesting that KM-00927 is a potential HDAC inhibitor. To investigate whether KM-00927 directly inhibited HDAC, an *in vitro* HDAC activity assay was performed. We found that SAHA, but not KM-00927 and rifabutin, effectively inhibited HDAC activity (figure 1*g*). Therefore, KM-00927 indirectly inhibits HDAC activity, leading to the acetylation of histones. However, the possibility cannot be excluded that KM-00927 is a prodrug and becomes active after entering a cell.

**Table 2.** Prediction of potential HDAC inhibitors by the LINCS. Differentially expressed genes (DEGs) induced by HDAC inhibitors were obtained from NCBI GEO database, including GSE22061 (SAHA- and FK228-treated HCT116 cells) and GSE60125 (SAHA-treated MDA-MB-231 cells). The full list of DEGs is shown in electronic supplementary material, table S1. These DEGs were inputted to the Lincscloud website and similar drugs were obtained.

ID	name	function	score	no. of cells	no. of signatures
BRD-K12867552	THM-I-94 (Abexinostat)	HDAC inhibitor	99.7612	9	15
BRD-K22503835	Scriptaid	HDAC inhibitor	98.9491	9	35
BRD-K81418486	Vorinostat (SAHA)	HDAC inhibitor	98.819	9	687
BRD-K69840642	ISOX (CAY10603)	HDAC6 inhibitor	98.7837	9	16
BRD-A19037878	Trichostatin-A	HDAC inhibitor	98.778	9	575
BRD-K02130563	Panobinostat (LBH589)	HDAC inhibitor	98.1105	9	68
BRD-K68202742	Trichostatin-A	HDAC inhibitor	97.8736	9	97
BRD-K64606589	Apicidin	HDAC inhibitor	97.4618	9	61
BRD-K17743125	Belinostat (PXD101)	HDAC inhibitor	97.3862	9	21
BRD-K13810148	Givinostat	HDAC inhibitor	96.8259	5	6
BRD-A39646320	HC-toxin	HDAC inhibitor	96.7645	9	21
BRD-K85493820	KM-00927	Unknown	96.7066	9	12
BRD-K56957086	Dacinostat (LAQ824)	HDAC inhibitor	96.597	9	23
BRD-K75081836	BRD-K75081836	unknown	96.2742	9	12
BRD-A94377914	Merck-ketone	HDAC inhibitor	96.2416	9	12
BRD-K52522949	NCH-51	HDAC inhibitor	95.5349	8	11

### 2.3. Integration of L1000 Fireworks Display (L1000FWD) into CMap/LINCS prediction

**Figure 2.**
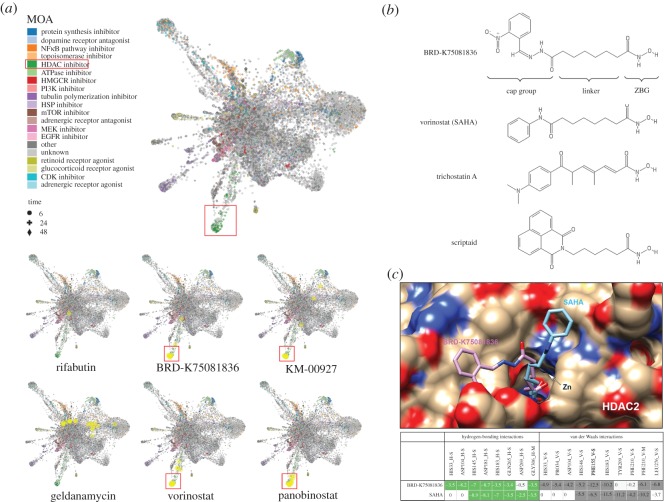
L1000FWD and molecular docking analyses of predicted HDAC inhibitors. (*a*) Upper part: gene signatures of drugs with known MOA were coloured and clustered. The red box indicated the cluster of HDAC inhibitors. Lower part: the gene signatures of drugs were queried online and visualized with the yellow circles. (*b*) The chemical structures of BRD-K75081836, vorinostat/SAHA, trichostatin A and scriptaid. (*c*) The upper part: the best docking model of BRD-K75081836 or SAHA to HDAC2. The lower part: the interaction residues of HDAC2 to BRD-K75081836 or SAHA.

Recently, an online Web tool, L1000 Fireworks Display (L1000FWD; http://amp.pharm.mssm.edu/L1000FWD), has been developed, which can provide visualization of drug-induced gene-expression signatures and their similarity in MOA [11]. As shown in figure 2*a* (the upper part), the advantage of L1000FWD is that gene signatures from different drug-treat cell types while sharing common MOA can be clustered together. Because CMap and LINCS analyses only gave predictions ranked according to the gene signature similarities, we thought that L1000FWD analysis may provide an additional improvement for CMap/LINCS predictions based on drugs' similarities in MOA. By querying L1000FWD for the predicted CMap and LINCS drugs (figure 1*a,d*), we found that gene signatures from cells treated with KM-00927 and BRD-K75081836 were clustered in the region of HDAC inhibitors, which was similar to the patterns of vorinostat/SAHA and panobinostat (highlighted in red boxes; figure 2*a*, the lower part) By contrast, gene signatures from cells treated with rifabutin or geldanamycin were not (figure 2*a*, the lower part). These results further support our results showing that KM-00927 is an HDAC inhibitor.

The above analyses also suggested that BRD-K75081836 may be a potent HDAC inhibitor. Actually, we found that the structure of BRD-K75081836 was very similar to hydroxamic acid-based HDAC inhibitors such as varinostat/SAHA, trichostatin A and scriptaid (figure 2*b*). Three structural moieties are typically found in hydroxamic acid-based HDAC inhibitors, including a surface recognition cap group that sits outside the active site of an HDAC enzyme and can tolerate extraordinary variability, a zinc-binding group (ZBG) that binds to the buried zinc ion within the active site, and between both, a linker fitting the tunnel of the active site [22]. To quantify the structural similarity between BRD-K75081836 and hydroxamic acid-based HDAC inhibitors, hierarchical clustering of drugs was performed using the ChemBioServer (http://chembioserver.vi-seem.eu/) [23]. Indeed, BRD-K75081836, but not other drugs (KM-00927, rifabutin and geldanamycin), was structurally similar to hydroxamic acid-based HDAC inhibitors including SAHA, trichostatin A, scriptaid and panobinostat. Among them, SAHA was the most similar one to BRD-K75081836 (electronic supplementary material, figure S1). Therefore, we hypothesized that BRD-K75081836 is a direct HDAC inhibitor. To support this notion, molecular docking analysis was performed to analyse the binding of BRD-K75081836 to the catalytic site of HDAC2. As shown in figure 2*c*, the binding modes of BRD-K75081836 and SAHA were similar, indicated by the overlapping hydrogen-bonding (green colour) and van der Waals (grey colour) interactions. In addition, the total fitness energy of BRD-K75081836 was higher than that of SAHA (table 3), suggesting the high binding affinity of BRD-K75081836 to HDAC2. These results support that BRD-K75081836 is a hydroxamic acid-based HDAC inhibitor, which warrants further investigation. Furthermore, we performed molecular docking of rifabutin and KM-00927 to HDAC2 in parallel. The results found that the total fitness energies of rifabutin and KM-00927 were lower than those of SAHA and BRD-K75081836 (table 3), further supporting the results that rifabutin and KM-00927 did not interact and inhibit HDAC activity *in vitro* (figure 1*g*).

**Table 3.** The interaction profile of HDAC2 and drugs by iGEMDOCK analysis. Fitness (kcal mol^−1^) is the total energy of a predicted pose in the binding site and it is calculated by the following equation: Fitness = VDW + Hbond + Elect. The terms, VDW, Hbond and Elect, indicate van der Waal energy, hydrogen bonding energy and Elect term is electro statistic energy, respectively.

drug	total fitness energy	VDW	Hbond	Elect
SAHA	−113.62	−80.11	−33.51	0
BRD-K75081836	−127.5	−85.63	−40.75	−1.12
Rifabutin	−96.35	−89.88	−6.47	0
KM-00927	−63.67	−59.38	−4.29	0

There are several types of HDAC inhibitors with efficacies in the nanomolar to millimolar ranges. The most potent types include cyclic peptides (such as depsipeptide/FK228, apicidin and trapoxin), hydroxomic acids (such as trichostatin A and SAHA) and benzamides (such as entinostat/MS-275 and mocetinostat), which inhibit HDAC activity in the nanomolar and low micromolar ranges and kill cancer cells in the sub-micromolar ranges. The ketones (such as α-ketomides and trifluoromethyl ketone) and short-chain fatty acids (such as valproic acid, phenylbutyrate and sodium butyrate) exhibit HDAC inhibition and cancer cell killing in the micromolar and millimolar ranges, respectively [24–26]. Despite the least potency of sort-chain fatty acid to inhibition of HDAC, some of them are used in clinic. For example, valproic acid is used to treat epilepsy and bipolar disorder [27,28]. In addition, valproic acid is still identified as a potent anti-cancer agent and encouraging results are found in clinical trials [29]. KM-00927 is a synthetic drug with unknown function. Our results found that KM-00927 was a potent and indirect HDAC inhibitor, which induced histone acetylation and killed cancer cells in micromolar ranges (10–50 µM). We thought that KM-00927 could be developed as an anti-cancer drug in the future. However, whether inhibition of HDAC activity contributes to its anti-cancer activity warrants further investigations.

### 2.4. Prediction of novel topoisomerase inhibitors by integrating the next-generation CMap and L1000FWD

Topoisomerases are enzymes that catalysed the winding and unwinding of DNA double helix through breaking the backbone of either one or both the DNA strands. There are two types of DNA topoisomerases, type I (subtypes: IA, IB and IC) and type II (subtypes: IIA and IIB), which break one or two strands of a DNA helix, respectively [30]. Topoisomerases are proved therapeutic targets of anti-cancer and antibacterial drugs. Clinically successful topoisomerase-targeting anti-cancer drugs act through topoisomerase poisoning, which leads to replication fork arrest and double-strand breaks [30]. Common topoisomerase I inhibitors include camptothecin and its derivatives such as topotecan, irinotecan and SN-38 (active metabolite of irinotecan). Examples of topoisomerase II inhibitors include etoposide, teniposide, doxorubicin and amsacrine.

**Figure 3.**
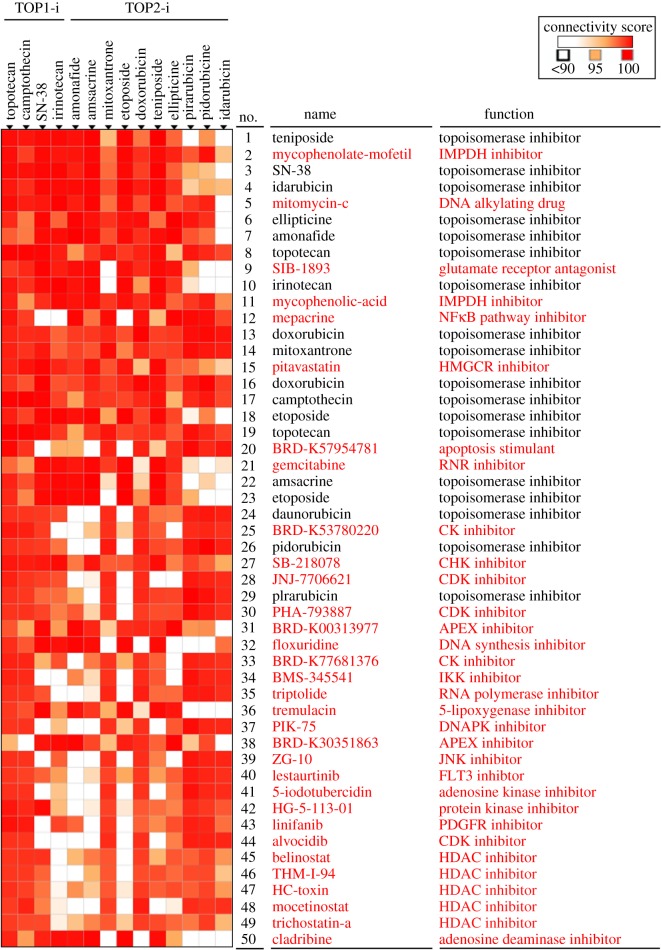
Prediction of novel topoisomerase inhibitors by the next-generation CMap. Connections of drug-gene signatures were analysed using the ‘Touchstone’ tool in the CLUE website (https://clue.io/). Connections were viewed as a heat map ranked by the summary connectivity score.

**Figure 4.**
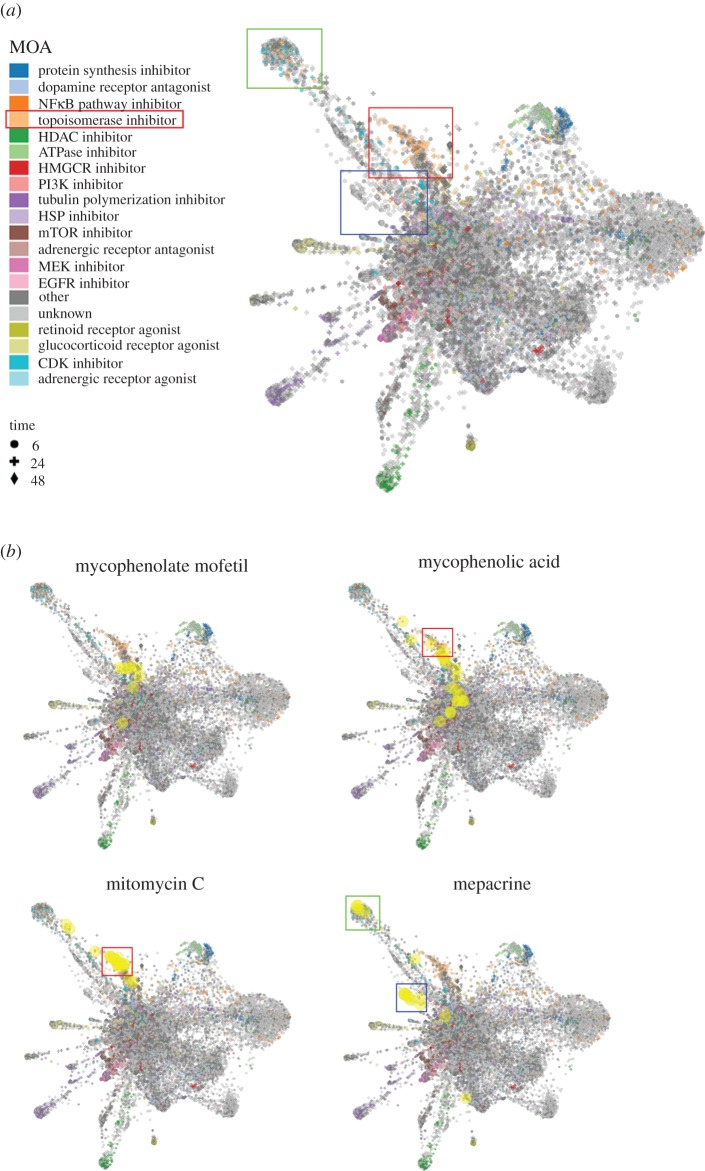
L1000FWD analysis of the predicted topoisomerase inhibitors by the next-generation CMap. (*a*) L1000FWD visualization of the drug-gene signatures. Drugs sharing similar MOA are clustered together. The red, green and blue boxes indicate the clusters of topoisomerase inhibitors. (*b*) L1000FWD visualization of mycophenolate mofetil, mycophenolic acid, mitomycin C and mepacrine.

To further support our drug repurposing strategy, analyses using the next-generation CMap and L1000FWD were performed to predict novel topoisomerase inhibitors. As shown in figure 3, 32 candidate drugs (highlighted in red) were obtained from top 50 perturbations that showed connections with topoisomerase I/II inhibitors. After L1000FWD analysis, we found that gene signatures of topoisomerase I/II inhibitors displayed three major clusters (figure 4*a*; electronic supplementary material, figure S2; highlighted in green, red and blue boxes). We chose mycophenolate mofetil, mycophenolic acid, mitomycin C and mepacrine for further experiments because of their higher ranking in the CLUE prediction (figure 3). Other candidate drugs with lower ranking were excluded because most of their gene signatures were overlapped with those of CDK inhibitors (electronic supplementary material, figure S3). Some drugs, such as SIB-1893, pitavastatin, gemcitabine and cladribine, have similar patterns with mycophenolate mofetil and mycophenolic acid (electronic supplementary material, figure S3; figure 4*b*). At the current stage, we only use mycophenolate mofetil/mycophenolic acid as a representative for further analysis.

**Figure 5.**
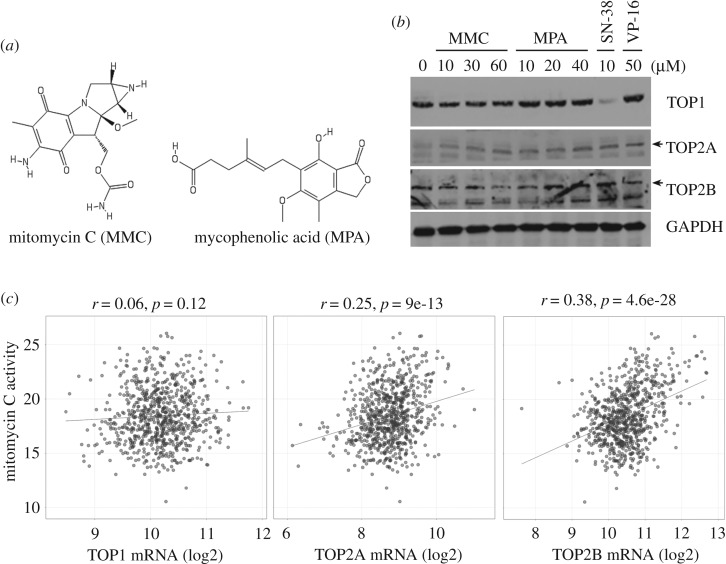
Effect of predicted topoisomerase inhibitors on the formation of DNA-topoisomerase cleavage complex. (*a*) The chemical structures of mycophenolic acid and mitomycin C. (*b*) HeLa cells were treated with the indicated concentration of drugs for 1 h. A band-depletion assay was performed as described in ‘Material and methods'. (*c*) The association between mitomycin C drug activity and topoisomerase expression was obtained by mining the CellMinerCDB (https://discover.nci.nih.gov/cellminercdb/).

Mycophenolate mofetil is a prodrug of mycophenolic acid that is an inhibitor of inosine monophosphate dehydrogenase (IMPDH). These two drugs are immunosuppressant drugs used to prevent rejection in organ transplantation [31]. Because only some gene signatures of mycophenolic acid and no gene signatures of mycophenolate mofetil were enriched in the clusters of topoisomerase inhibitors (figure 4*b*), we speculated that both mycophenolate mofetil and mycophenolic acid may not be topoisomerase inhibitors. Thus, mycophenolic acid was used as a negative example for validation. Mitomycin C, a DNA alkylating agent, has been widely used as chemotherapy treatment for various cancer types [32]. Mepacrine, also called quinacrine, is an anti-malarial drug. Both mitomycin C and mepacrine had gene signatures enriched in the clusters of topoisomerase inhibitors (figure 4*b*), indicating that they may be potential topoisomerase inhibitors. Indeed, mepacrine has been shown to display anti-cancer activity through inhibition of topoisomerase activity [33]. Therefore, we further examined and compared the effects of mitomycin C and mycophenolic acid (figure 5*a*). Topoisomerase activity was measured by a band-depletion assay that detects the presence of catalytic topoisomerase-DNA cleavage complexes trapped by topoisomerase inhibitors. Topoisomerase-DNA complexes have lower mobility during SDS-PAGE compared to free enzymes [34]. As shown in figure 5*b*, mitomycin C, but not mycophenolic acid, depleted the band of topoisomerase IIB (TOP2B), but not topoisomerase 1 (TOP1) and IIA (TOP2A). As positive controls, SN-38 and etoposide (VP-16) depleted TOP1 and TOP2B, respectively. Therefore, mitomycin C acts like etoposide and is a topoisomerase IIB inhibitor.

It has been shown that intrinsic sensitivity of cancer cells to topoisomerase II inhibitors is positively and highly correlated with the topoisomerase II expression [35–37]. To investigate whether the cell-killing activity of mitomycin C was associated with topoisomerase II expression, drug sensitivity and gene-expression data were obtained by querying the Cancer Therapeutics Response Portal (CTRP [38–40]) using the CellMinerCDB (https://discover.nci.nih.gov/cellminercdb/) [41]. As shown in figure 5*c*, drug activity of mitomycin C was positively correlated with both *TOP2A* and *TOP2B*, but not *TOP1* mRNA expression. These results supported that topoisomerase II expression could be used as a therapeutic biomarker for mitomycin C.

To investigate the interaction between mitomycin C and topoisomerase IIB, molecular docking was performed. As shown in electronic supplementary material, table S2, the binding affinity of mitomycin C to topoisomerase IIB was lower than that of etoposide, suggesting that additional mechanism(s) might be involved in the action of mitomycin C. An enzymatic bioreduction of mitomycin C is required for its activation and cytotoxic effect. Upon reduction, mitomycin C is converted into a highly bis-electrophilic intermediate that alkylates and crosslinks DNA [42]. Mitomycin C alkylations are specific for the guanine nucleosides in the CpG sequences [43]. It has been shown that psorospermin, a plant-derived anti-tumour agent, alkylates the guanine at the topoisomerase II cleavage site to trap the topoisomerase II-DNA cleavage complex [44]. In addition, clerocidin, a topoisomerase II poison, alkylates and forms covalent adducts with guanines, then trapping topoisomerase II enzymes [45]. These studies support the action of mitomycin C to promote the formation of topoisomerase II-DNA cleavage complex (figure 5*b*). Therefore, our results provide an additional mechanistic insight into the mode of action of the anti-cancer agent mitomycin C, which may extend its clinical application for treating cancers or other diseases. However, the exact mechanism warrants further investigation.

## 3. Conclusion

**Figure 6.**
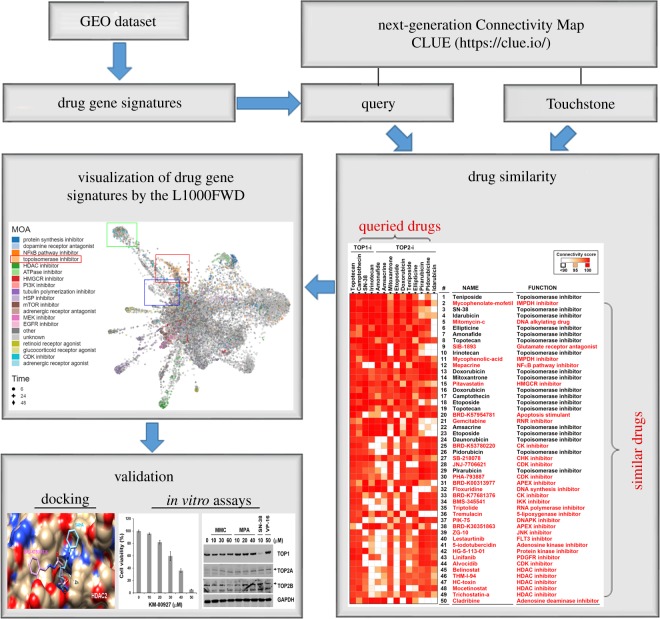
A workflow for the strategy of drug repurposing and drug MOA prediction.

More and more large-scale biological databases and user-friendly analytic tools are developed, allowing scientists to obtain comprehensive and systematic insights into the scientific problems, even those without a computational background. This study is a prime example of utilization and integration of these freely available public resources. We found that combined analyses using L1000-based CMap/LINCS and L1000FWD provide reliable prediction for drug repurposing and investigation of drug MOA. According to our analysis, a workflow is therefore proposed (figure 6). The gene signatures of drugs can be either prepared from GEO datasets or directly obtained by querying the CLUE database using the ‘Touchstone’ tool. Similar drugs can be further compared by the L1000FWD visualization of gene signatures to select candidates sharing similar MOAs. Finally, candidate drugs are validated by molecular docking and *in vitro* assays. Based on this strategy, this study provides two proof-of-concept examples and identifies novel HDAC inhibitors (KM-00927 and BRD-K75081836) and topoisomerase IIB inhibitor (mitomycin C). The anti-cancer activity and MOA of these drugs will be investigated in detail in the future.

## 4. Material and methods

### 4.1. Connectivity Map analysis

For the CMap build 02 analyses, the DEGs (electronic supplementary material, File S1) from HDAC inhibitor-treated cancer cell lines, including SAHA-treated HCT116 (GSE22061 [20]), SAHA-treated MDA-MB-231 (GSE60125) and FK228-treated HCT116 cells (GSE22061 [20]), were prepared using the GEO online tool GEO2R (https://www.ncbi.nlm.nih.gov/geo/geo2r/) [9]. GEO2R performs comparisons on original submitter-supplied processed data tables using the GEOquery and limma R packages from the Bioconductor project [9]. The cut-off criteria for DEGs were set at adjusted *p*-value <0.01 and |logFC|> 2. Gene symbols were converted into probe IDs of the Affymetrix GeneChip Human Genome U133A Array (HG-U133A) by an online tool, the NetAffx (https://www.affymetrix.com/analysis/index.affx; Affymetrix). Then, these probe sets were queried according to the instruction. For the LINCS analyses, theses DEGs (gene symbols) were directly inputted in the Lincscloud website (http://www.lincscloud.org/). It should be noted that the Lincscloud website has been deprecated while this study was in progress. For the next-generation CMap (the CLUE) analyses, the online ‘Touchstone’ tool was used to directly explore the connections of gene signatures of drugs. For the prediction of novel topoisomerase inhibitors, the ‘perturbation classes' and ‘perturbation type’ were set as ‘CMap class’ and ‘compound’, respectively. Drugs with description ‘topoisomerase inhibitor’ were selected for generation of a heat map (figure 3). The drug similarity was ranked according to the CMap connectivity score (ranging from −100 to 100) that corresponds to the fraction of reference gene sets with a greater similarity to the perturbagen than the current query. For example, a score of 95 indicates that only 5% of reference gene sets showed stronger connectivity than the current query to the perturbagen. In general, connectivity scores higher than 95 and lower than −95 were considered as strong scores [7]. For L1000FWD analysis, drug data can be queried and accessed by inputting drug name on the website (http://amp.pharm.mssm.edu/L1000FWD/). Drugs sharing similar MOAs will be clustered together [11].

### 4.2. Chemical structure similarity analysis by the ChemBioServer

The ChemBioServer is a Web application for filtering, clustering and visualizing of chemical compounds [23]. For the hierarchical clustering of compounds, a SDF file containing the simplified molecular-input line-entry system (SMILES) of drugs was uploaded to the ChemBioServer. The parameters were set as follows: distance = Soergel (Tanimoto coefficient); clustering linkage = Ward; and clustering threshold = 0.4.

### 4.3. Molecular docking

Docking simulations were performed with iGEMDOCK [46]. The structures of HDAC2 (4LXZ) and topoisomerase IIB (3QX3) were obtained from the Protein Data Bank. The 3D drug structures in MOL2 format were prepared using the software, Open Babel v. 2.4.1 [47]. The binding site of the target was prepared, and energy-minimized compounds were imported. The docking protocol is set to 80 generations per ligand and a population size of 300 random individuals. All docking conformations were performed 10 times using a genetic evolutionary algorithm, and the fitness of the docked structures was calculated. The binding site for docking was defined by the bound ligand included in the downloaded HDAC2 protein structure, and the binding site radius was set to 8 Å. The binding affinity of a drug to HDAC2 was estimated by the fitness value calculated via the following formula: Fitness (kcal mol^−1^) = VDW + Hbond + Elect, where the VDW term is van der Waal energy, Hbond term is hydrogen-bonding energy and Elect term is electro statistic energy.

### 4.4. Materials

McCoy's 5A medium, RPMI-1640 medium, DMEM medium, L-glutamine, sodium pyruvate and antibiotic-antimycotic (penicillin G, streptomycin and amphotericin B) were purchased from Life Technologies. Fetal bovine serum (FBS) was purchased from Biological Industries. Acetyl histones H3 and H4 antibodies were purchased from MerckMillipore. Topoisomerases I (TOP1), IIA (TOP2A) and IIB (TOP2B), and GAPDH antibodies were purchased from GeneTex. Horseradish peroxidase-labelled goat anti-rabbit and anti-mouse secondary antibodies were purchased from Jackson ImmunoResearch. KM-00927 was purchased from MolPort. Mitomycin C was purchased from APExBio. Suberoylanilide hydroxamic acid (SAHA) was purchased from Cayman. Rifabutin, DMSO and 3-(4,5-dimethylthiazol-2-yl)-2,5-diphenyl tetrazolium bromide (MTT) were purchased from Sigma. Protease and phosphatase inhibitor cocktails were purchased from Roche.

### 4.5. Cell culture

HeLa human cervical cancer cells were cultured in RPMI-1640 medium. HCT116 human colorectal cancer cells were cultured in McCoy's 5A medium. The culture medium was supplemented with 10% FBS, 2 mM L-glutamine, 1 mM sodium pyruvate, 1% antibiotic-antimycotic. Cells were cultured at 37°C and 5% CO_2_ in a humidified incubator and passaged every 2 or 3 days.

### 4.6. Cell viability assay

Cell viability was measured by an MTT assay. Cells (3–5 × 10^3^/well) were plated in 96-well plates and treated with drugs for 72 h. Then, 0.5 mg ml^−1^ MTT was added. After 4 h, the blue MTT formazan precipitates were dissolved in 200 µl DMSO. The absorbance at 550 nm was measured on a multi-well plate reader.

### 4.7. *In vitro* HDAC activity assay

The global HDAC activity was determined with an HDAC Activity Colorimetric Assay Kit (BioVision). Nuclear extracts were prepared from HeLa or HCT116 cells by the Nuclear/Cytosol Fractionation Kit (BioVision). Incubations were performed at 37°C with nuclear extracts (10 μg) and drugs (KM-00927, rifabutin or SAHA), and the HDAC reaction was initiated by adding the Ac-Lys(Ac)-pNA substrate. After 1 h, Lysine Developer was added to stop the reaction, and the mixture was incubated for another 30 min. The optical density (OD) at 405 nm was measured using a multi-well plate reader. HDAC activity was expressed as the relative OD value.

### 4.8. Western blot analysis

Cells were lysed in ice-cold buffer containing 50 mM Tris–HCl (pH 7.5), 150 mM NaCl, 1 mM MgCl_2_, 2 mM EDTA, 1% NP-40, 10% glycerol, 1 mM DTT and 1× protease and phosphatase inhibitor cocktails at 4°C for 30 min. After centrifugation, the supernatants were transferred to new tubes and stored at −20°C. Protein concentrations were determined with a Bio-Rad Protein Assay. Proteins in the cell lysate (30–50 µg) were separated on a 4–15% sodium dodecylsulfate-polyacrylamide gradient gel, then transferred electrophoretically onto nitrocellulose membranes (Bio-Rad). Membranes were pre-hybridized 5% skim milk in TBST buffer (20 mM Tris-base, 150 mM NaCl, 0.05% Tween-20) for 1 h, then transferred to 1% BSA/TBST containing a primary antibody. After incubation overnight at 4°C, membranes were washed with TBST buffer and then submerged in 1% BSA/TBST containing a horseradish peroxidase-conjugated secondary antibody for 1 h. Membranes were washed with TBST buffer, then developed using the Western Lightning Plus ECL detecting reagent (PerkinElmer) and exposed to X-ray film (Fujifilm).

### 4.9. Band-depletion assay

Cells (5 × 10^5^/dish) were seeded in 60 mm dishes and treated with drugs for 1 h. Then, cells were immediately lysed with 1× SDS sample buffer (150 µl). After vortexing and boiling at 100°C for 5 min, protein was separated by 7.5% SDS-PAGE and then topoisomerase expression was analysed by Western blot analysis.

## Supplementary Material

Supplementary data

## Data accessibility

The datasets supporting this article have been uploaded as part of the electronic supplementary material.
