# Supplementary material for: Systematic polypharmacology and drug repurposing via an integrated L1000-based Connectivity Map database mining
Source: R Soc Open Sci. 2018 Nov 28;5(11):181321. doi: 10.1098/rsos.181321 (PMC6281908; doi:10.1098/rsos.181321)
Supplement: Supplementary data [file rsos181321supp1.pdf]

Supplementary Information

Systematic polypharmacology and drug repurposing via an integrated L1000-based Connectivity Map database mining

Tsang-Pai Liu[1,2,3,4,5,#], Yao-Yu Hsieh[1,6,7,#], Chia-Jung Chou[1,8,9], Pei-Ming Yang[1,8,9,*]

[1] *Ph.D. Program for Cancer Molecular Biology and Drug Discovery, College of Medical Science and Technology, Taipei Medical University and Academia Sinica, Taipei, Taiwan*
[2] *Department of Surgery, Mackay Memorial Hospital, Taipei, Taiwan*
[3] *Mackay Junior College of Medicine, Nursing and Management, New Taipei City, Taiwan*
[4] *Department of Medicine, Mackay Medical College, New Taipei City, Taiwan*
[5] *Liver Medical Center, Mackay Memorial Hospital, Taipei, Taiwan*
6 Division of Hematology and Oncology, Shuang Ho Hospital, Taipei Medical University, New Taipei City, Taiwan
7 Division of Hematology and Oncology, Department of Internal Medicine, School of Medicine, College of Medicine, Taipei Medical University
[8] *Graduate Institute of Cancer Biology and Drug Discovery, College of Medical Science and Technology, Taipei Medical University, Taipei, Taiwan*
[9] *TMU Research Center of Cancer Translational Medicine*

[#] Equal contribution.
[*] Address correspondence to: Dr. Pei-Ming Yang, Graduate Institute of Cancer Biology and Drug Discovery, College of Medical Science and Technology, Taipei Medical University, 250 Wu-Hsing Street, Taipei 11031, Taiwan. Tel: +886-2-27361661 ext. 7629; Fax: +886-2-26558562; E-mail: yangpm@tmu.edu.tw; orcid.org/0000-0002-4004-2518

Supplementary Information includes:
Supplementary Figures S1-S3
Supplementary Tables S1 and S2

Legends to Supplementary Figures

Figure S1. Chemical structure similarity analysis. A SDF files containing the simplified molecular-input line-entry system (SMILES) of drugs was uploaded to the ChemBioServer for the hierarchical clustering of compounds. The parameters were set as follows: distance = Soergel (Tanimoto coefficient); clustering linkage = Ward; and clustering threshold = 0.4. The compounds in red grids indicated that they were classified in the same clusters with clustering threshold less than 0.4.

Figure S2. L1000FWD visualization of the drug gene signatures for topoisomerase inhibitors. Drugs sharing similar MOA were clustered together. The red, green, and blue boxes indicated the major clusters of topoisomerase inhibitors.

Figure S3. L1000FWD visualization of the drug gene signatures for the predicted topoisomerase inhibitors. Drugs sharing similar MOA were clustered together.

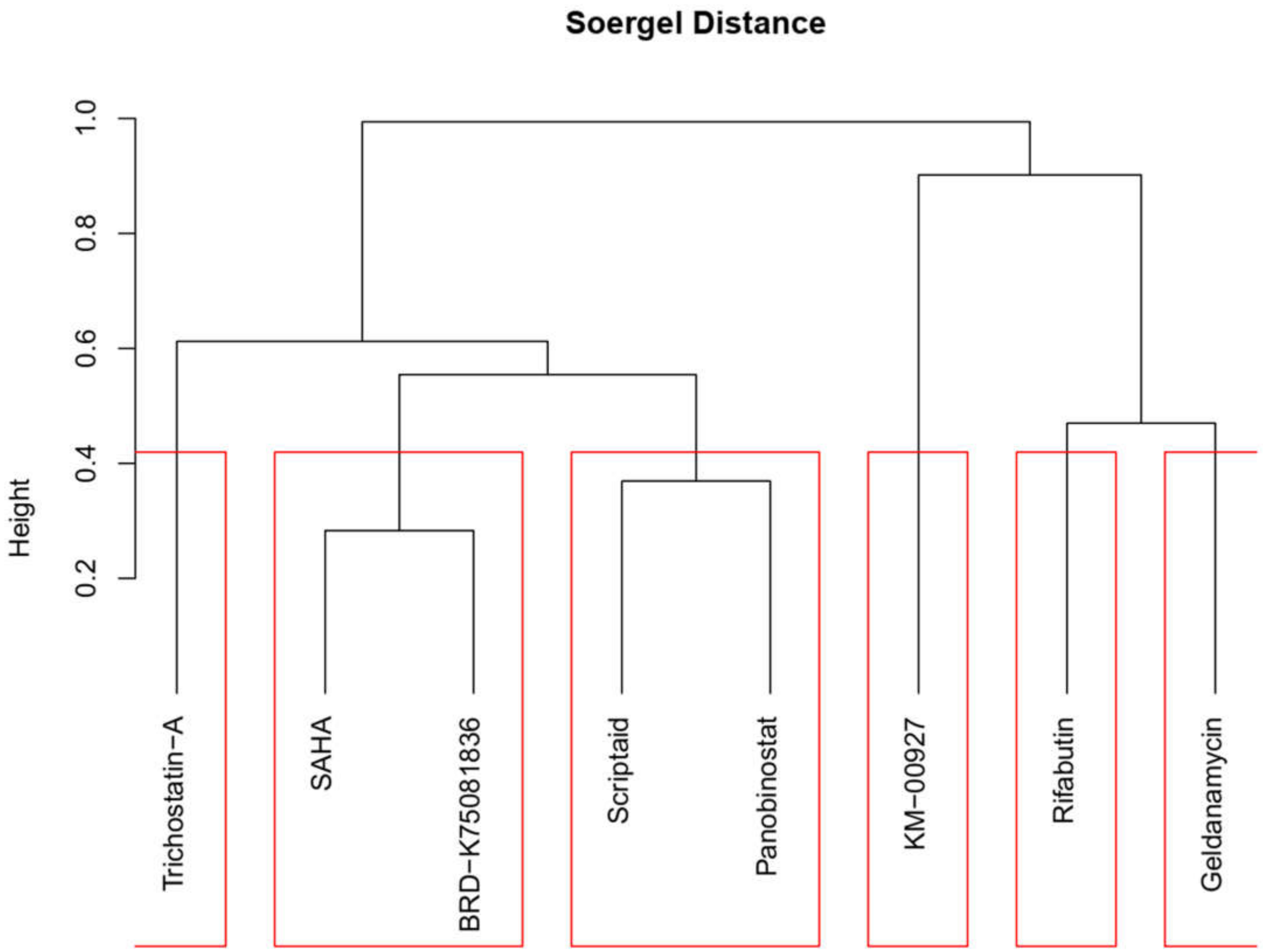
Soergel Distance
Height
1.0
0.8
0.6
0.4
0.2
Trichostatin−A
SAHA
BRD−K75081836
Scriptaid
Panobinostat
KM−00927
Rifabutin
Geldanamycin

Suppl. Fig. S1

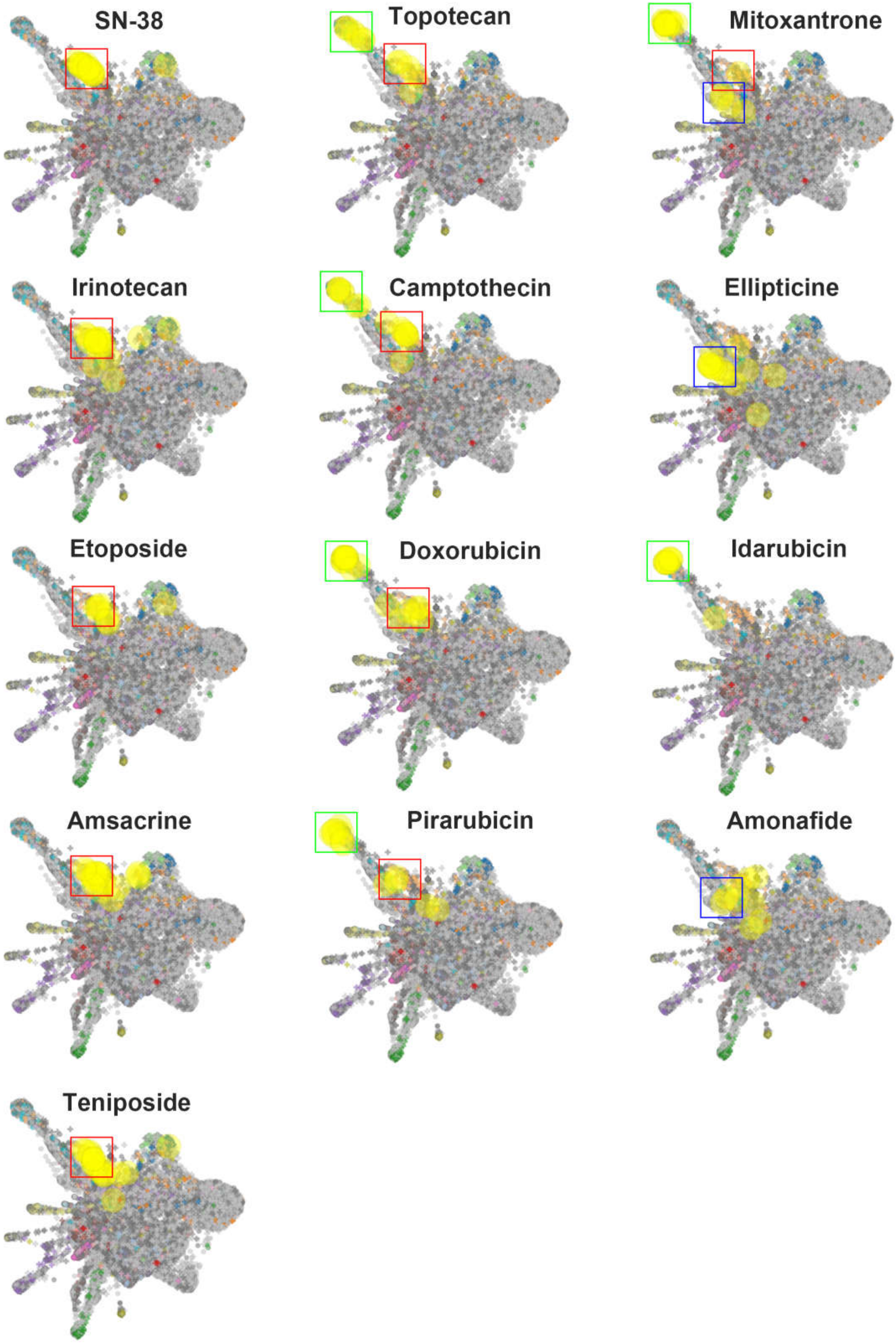
SN-38
Topotecan
Mitoxantrone
Irinotecan
Camptothecin
Ellipticine
Etoposide
Doxorubicin
Idarubicin
Amsacrine
Pirarubicin
Amonafide
Teniposide

Suppl. Fig. S2

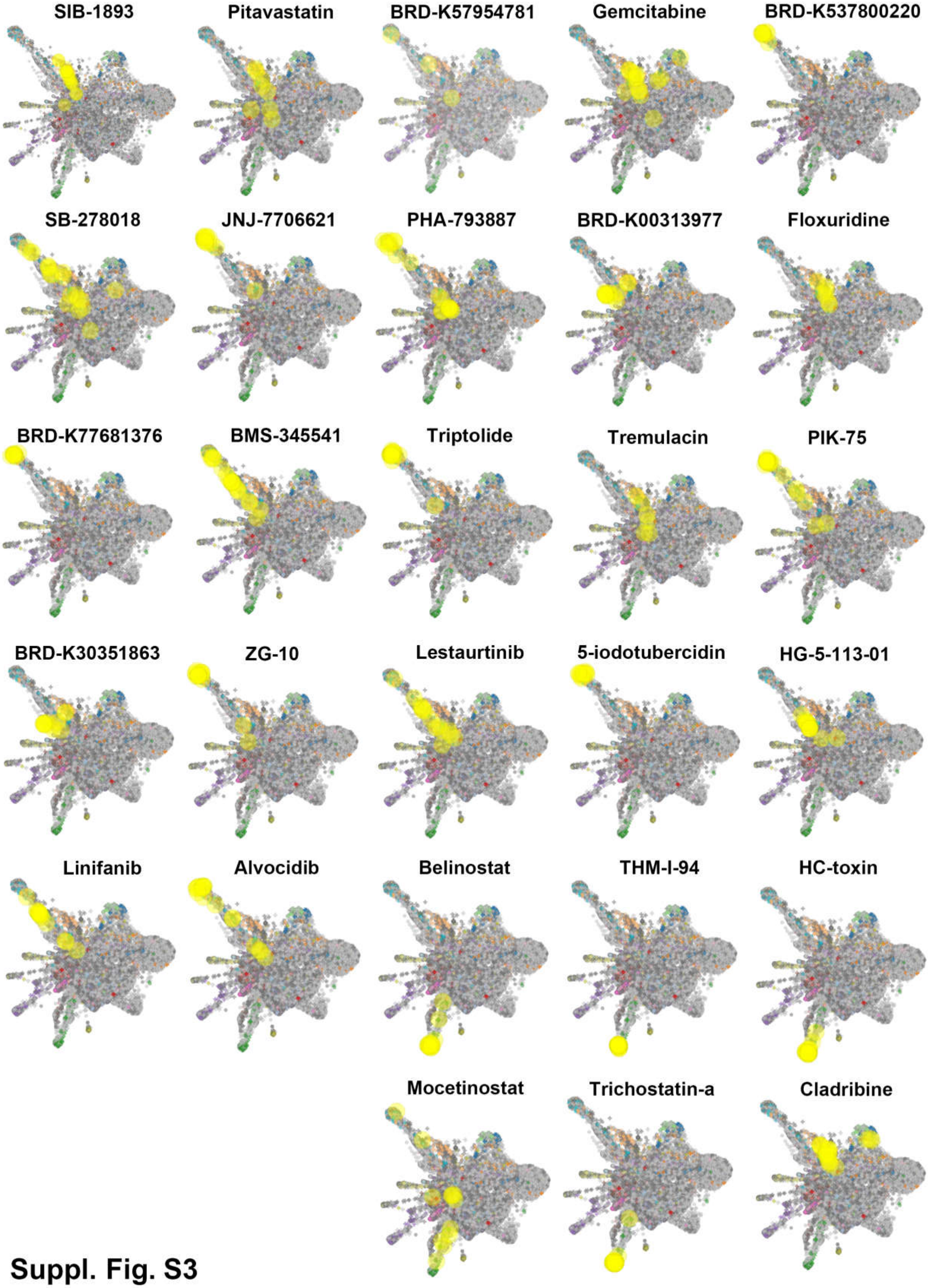

SIB-1893
Pitavastatin
BRD-K57954781
Gemcitabine
BRD-K537800220
SB-278018
JNJ-7706621
PHA-793887
BRD-K00313977
Floxuridine
BRD-K77681376
BMS-345541
Triptolide
Tremulacin
PIK-75
BRD-K30351863
ZG-10
Lestaurtinib
5-iodotubercidin
HG-5-113-01
Linifanib
Alvocidib
Belinostat
THM-I-94
HC-toxin
Mocetinostat
Trichostatin-a
Cladribine

Suppl. Fig. S3

Table S1. Differentially expressed genes (DEGs) from HDAC inhibitor-treated cancer cells. DEGs from all datasets were prepared by GEO2R web tool. The cut-off criteria were set at adjusted p value < 0.01 and |logFC| > 2.

GSE22061_HCT116-SAHA			GSE22061_HCT116-FK228			GSE60125_MDA-MB-231-SAHA		
ID	logFC	Gene.symbol	ID	logFC	Gene.symbol	ID	logFC	Gene.symbol
214079_at	4.87	DHRS2	206463_s_at	5.09	DHRS2	215704_at	5.88	FLG
206463_s_at	4.84	DHRS2	209197_at	4.92	SYT11	1555673_at	5.23	KRTAP2-3
202149_at	4.01	NEDD9	214079_at	4.89	DHRS2	227803_at	4.55	ENPP5
213139_at	3.65	SNAI2	202149_at	4.18	NEDD9	227140_at	4.31	INHBA
44783_s_at	3.52	HEY1	209198_s_at	4.03	SYT11	214079_at	3.99	DHRS2
203787_at	3.32	SSBP2	227812_at	4.02	TNFRSF19	233504_at	3.96	C9orf84
227354_at	3.3	PAG1	221541_at	3.99	CRISPLD2	203980_at	3.92	FABP4
227812_at	3.3	TNFRSF19	213139_at	3.98	SNAI2	210511_s_at	3.86	INHBA
228715_at	3.3	ZCCHC12	222449_at	3.96	PMEPA1	213931_at	3.85	ID2B
209392_at	3.28	ENPP2	210095_s_at	3.91	IGFBP3	228865_at	3.79	C1orf116
225626_at	3.25	PAG1	209392_at	3.87	ENPP2	206785_s_at	3.74	KLRC2
232165_at	3.18	EPPK1	44783_s_at	3.82	HEY1	206569_at	3.72	IL24
217028_at	3.16	CXCR4	209101_at	3.74	CTGF	201744_s_at	3.71	LUM
208092_s_at	3.16	FAM49A	224225_s_at	3.67	ETV7	204515_at	3.6	HSD3B1
225622_at	3.11	PAG1	203787_at	3.65	SSBP2	209459_s_at	3.56	ABAT
232164_s_at	3.08	EPPK1	208436_s_at	3.65	IRF7	1559336_at	3.54	LOC101928505
218559_s_at	3.08	MAFB	232165_at	3.64	EPPK1	217979_at	3.54	TSPAN13
210095_s_at	3.04	IGFBP3	1570445_a_at	3.56	LOC643201	228221_at	3.52	SLC44A3
205003_at	2.92	DOCK4	217028_at	3.54	CXCR4	207102_at	3.37	AKR1D1
226811_at	2.92	FAM46C	226811_at	3.53	FAM46C	266_s_at	3.37	CD24
201109_s_at	2.91	THBS1	225626_at	3.51	PAG1	1552365_at	3.29	SCIN
209683_at	2.88	FAM49A	228846_at	3.48	MXD1	215785_s_at	3.25	CYFIP2
221541_at	2.87	CRISPLD2	206465_at	3.48	ACSBG1	219874_at	3.24	SLC12A8
209101_at	2.87	CTGF	206619_at	3.45	DKK4	219702_at	3.22	PLAC1
218087_s_at	2.86	SORBS1	218559_s_at	3.45	MAFB	231930_at	3.21	ELMOD1
218839_at	2.84	HEY1	227354_at	3.44	PAG1	216379_x_at	3.2	CD24
222449_at	2.82	PMEPA1	232164_s_at	3.42	EPPK1	208650_s_at	3.19	CD24
209197_at	2.8	SYT11	209189_at	3.39	FOS	243610_at	3.17	C9orf135
226281_at	2.78	DNER	225622_at	3.37	PAG1	206145_at	3.17	RHAG
223484_at	2.77	C15orf48	228228_at	3.37	DACT3	209771_x_at	3.14	CD24
208156_x_at	2.76	EPPK1	226275_at	3.34	MXD1	235230_at	3.14	PLCXD2
228846_at	2.75	MXD1	214414_x_at	3.31	HBA2	204035_at	3.12	SCG2
214414_x_at	2.69	HBA2	1569144_a_at	3.31	CYSRT1	203498_at	3.09	RCAN2
225283_at	2.68	ARRDC4	218839_at	3.21	HEY1	220921_at	3.09	SPANXB1
206100_at	2.68	CPM	212843_at	3.20	NCAM1	242162_at	3.08	DAW1
209189_at	2.68	FOS	209683_at	3.19	FAM49A	210145_at	3.08	PLA2G4A
222513_s_at	2.66	SORBS1	228715_at	3.18	ZCCHC12	241703_at	3.08	RUNDC3B
238532_at	2.61	DPF3	209568_s_at	3.12	RGL1	215321_at	3.07	RUNDC3B
204646_at	2.61	DPYD	210829_s_at	3.12	SSBP2	207733_x_at	3.06	PSG9
224559_at	2.6	MALAT1	218087_s_at	3.09	SORBS1	205352_at	3.06	SERPINI1
212843_at	2.6	NCAM1	208926_at	3.07	NEU1	1557636_a_at	3.05	C7orf57
208926_at	2.59	NEU1	223913_s_at	3.06	MIR7-3HG	205569_at	3.02	LAMP3
210829_s_at	2.58	SSBP2	208156_x_at	3.04	EPPK1	213050_at	3.01	COBL
209839_at	2.56	DNM3	208092_s_at	3.04	FAM49A	219476_at	3	C1orf116
225540_at	2.51	MAP2	227394_at	3.03	NCAM1	209594_x_at	3	PSG9
209459_s_at	2.5	ABAT	205493_s_at	3.02	DPYSL4	229242_at	3	TNFSF15
226275_at	2.49	MXD1	222513_s_at	2.97	SORBS1	209738_x_at	2.96	PSG6
209160_at	2.47	AKR1C3	205372_at	2.95	PLAG1	230147_at	2.94	F2RL2
213256_at	2.47	MARCH3	201109_s_at	2.93	THBS1	223821_s_at	2.94	SUSD4
201110_s_at	2.46	THBS1	222450_at	2.91	PMEPA1	231504_at	2.93	CCDC148
224225_s_at	2.45	ETV7	201627_s_at	2.91	INSIG1	209392_at	2.9	ENPP2
235334_at	2.45	ST6GALNAC3	209201_x_at	2.90	CXCR4	238778_at	2.88	MPP7
227394_at	2.44	NCAM1	238532_at	2.86	DPF3	206907_at	2.88	TNFSF9
203498_at	2.44	RCAN2	227909_at	2.86	LINC00087	220468_at	2.86	ARL14
229518_at	2.43	FAM46B	209083_at	2.83	CORO1A	236984_at	2.85	C4orf26
231166_at	2.43	GPR155	226487_at	2.81	FAM222A	203108_at	2.81	GPRC5A
210971_s_at	2.41	ARNTL	205807_s_at	2.77	TUFT1	227399_at	2.81	VGLL3
228570_at	2.41	BTBD11	229518_at	2.77	FAM46B	204830_x_at	2.8	PSG5

227209_at	2.41	CNTN1	220505_at	2.75	C9orf53	206204_at	2.78	GRB14
228228_at	2.4	DACT3	211919_s_at	2.74	CXCR4	211741_x_at	2.77	PSG3
226487_at	2.36	FAM222A	231166_at	2.72	GPR155	203399_x_at	2.76	PSG3
204446_s_at	2.34	ALOX5	204595_s_at	2.71	STC1	223233_s_at	2.75	CGN
209568_s_at	2.34	RGL1	209459_s_at	2.70	ABAT	206561_s_at	2.73	AKR1B10
235683_at	2.34	SESN3	226281_at	2.67	DNER	220922_s_at	2.72	SPANXA2
211919_s_at	2.31	CXCR4	209460_at	2.67	ABAT	235228_at	2.71	CCDC85A
209201_x_at	2.31	CXCR4	205003_at	2.66	DOCK4	228796_at	2.7	CPNE4
205372_at	2.31	PLAG1	1566840_at	2.63	LOC283674	206191_at	2.7	ENTPD3
1558501_at	2.3	DNM3	230547_at	2.62	KCNC1	206295_at	2.7	IL18
205352_at	2.3	SERPINI1	222248_s_at	2.61	SIRT4	208191_x_at	2.69	PSG4
228645_at	2.28	SNHG9	213256_at	2.59	MARCH3	231947_at	2.68	MYCT1
206465_at	2.27	ACSBG1	204597_x_at	2.59	STC1	219410_at	2.68	TMEM45A
201627_s_at	2.27	INSIG1	206100_at	2.58	CPM	223609_at	2.67	ROPN1L
223217_s_at	2.27	NFKBIZ	236442_at	2.58	DPF3	225809_at	2.65	PARM1
1569144_a_at	2.25	CYSRT1	201626_at	2.57	INSIG1	201107_s_at	2.64	THBS1
228062_at	2.24	NAP1L5	209839_at	2.55	DNM3	225987_at	2.61	STEAP4
204072_s_at	2.22	FRY	200664_s_at	2.54	DNAJB1	229430_at	2.6	C8orf46
212774_at	2.22	ZBTB18	222921_s_at	2.52	HEY2	223878_at	2.6	INPP4B
227909_at	2.2	LINC00087	227283_at	2.51	EFR3B	205602_x_at	2.59	PSG7
205807_s_at	2.2	TUFT1	228570_at	2.51	BTBD11	233576_at	2.58	HMGCLL1
209460_at	2.19	ABAT	201625_s_at	2.51	INSIG1	213782_s_at	2.58	MYOZ2
238692_at	2.19	BTBD11	234989_at	2.48	NEAT1	205239_at	2.56	AREG
227283_at	2.19	EFR3B	225283_at	2.48	ARRDC4	236787_at	2.54	LOC100507286
235019_at	2.16	CPM	235334_at	2.47	ST6GALNAC3	238681_at	2.52	GDPD1
205259_at	2.16	NR3C2	234513_at	2.47	ELOVL3	202800_at	2.52	SLC1A3
204917_s_at	2.15	MLLT3	238692_at	2.46	BTBD11	205513_at	2.52	TCN1
205586_x_at	2.15	VGF	210240_s_at	2.46	CDKN2D	227919_at	2.51	UCA1
243087_at	2.14	WDR63	223217_s_at	2.45	NFKBIZ	208134_x_at	2.5	PSG2
1554440_at	2.13	KIAA0513	205294_at	2.45	BAIAP2	237131_at	2.5	RIIAD1
223218_s_at	2.13	NFKBIZ	230104_s_at	2.43	TPPP	220327_at	2.5	VGLL3
1554178_a_at	2.1	FAM126B	205100_at	2.39	GFPT2	206290_s_at	2.48	RGS7
216060_s_at	2.09	DAAM1	220444_at	2.38	ZNF557	215821_x_at	2.45	PSG3
209083_at	2.08	CORO1A	1554178_a_at	2.38	FAM126B	219263_at	2.43	RNF128
222456_s_at	2.08	LIMA1	223484_at	2.35	C15orf48	231166_at	2.42	GPR155
210657_s_at	2.08	SEP4	201110_s_at	2.35	THBS1	222549_at	2.41	CLDN1
204918_s_at	2.07	MLLT3	1558501_at	2.35	DNM3	1555538_s_at	2.4	FAM9B
242814_at	2.07	SERPINB9	219529_at	2.34	CLIC3	1564757_a_at	2.39	CCDC148
221563_at	2.06	DUSP10	235683_at	2.34	SESN3	238451_at	2.39	MPP7
201626_at	2.05	INSIG1	230900_at	2.34	CCDC110	228523_at	2.39	NANOS1
208436_s_at	2.04	IRF7	204411_at	2.34	KIF21B	205767_at	2.38	EREG
209993_at	2.03	ABCB1	224559_at	2.33	MALAT1	235362_at	2.36	LOC729970
231472_at	2.03	FBXO15	203498_at	2.33	RCAN2	218087_s_at	2.36	SORBS1
230900_at	2.02	CCDC110	225540_at	2.31	MAP2	227862_at	2.36	TRNP1
210105_s_at	2.01	FYN	202627_s_at	2.31	SERPINE1	223315_at	2.34	NTN4
201739_at	2.01	SGK1	214440_at	2.31	NAT1	208257_x_at	2.33	PSG1
228063_s_at	2	NAP1L5	231874_at	2.31	FAM126B	205709_s_at	2.32	CDS1
204595_s_at	2	STC1	210839_s_at	2.31	ENPP2	225540_at	2.32	MAP2
230252_at	-2	LPAR5	218642_s_at	2.31	CHCHD7	208651_x_at	2.31	CD24
204619_s_at	-2	VCAN	206340_at	2.30	NR1H4	241986_at	2.3	BMPER
222819_at	-2.01	CTPS2	227209_at	2.30	CNTN1	231618_s_at	2.29	SUN3
220615_s_at	-2.01	FAR2	225855_at	2.29	EPB41L5	203543_s_at	2.28	KLF9
242005_at	-2.01	LINC00973	204457_s_at	2.27	GAS1	211756_at	2.28	PTHLH
210993_s_at	-2.01	SMAD1	204646_at	2.27	DPYD	1568970_at	2.27	ADAM18
209651_at	-2.01	TGFB1I1	201739_at	2.26	SGK1	235606_at	2.27	LINC00883
235124_at	-2.02	EIF3J-AS1	227868_at	2.26	LOC154761	207148_x_at	2.27	MYOZ2
221935_s_at	-2.02	EOGT	226913_s_at	2.26	SOX8	203413_at	2.27	NELL2
1555881_s_at	-2.02	LZTS2	220302_at	2.25	MAK	1557132_at	2.27	WDR17
228280_at	-2.03	ZC3HAV1L	223218_s_at	2.25	NFKBIZ	206029_at	2.26	ANKRD1
220617_s_at	-2.03	ZNF532	1563053_at	2.25	LOC729083	225283_at	2.25	ARRDC4
225181_at	-2.04	ARID1B	204446_s_at	2.24	ALOX5	227209_at	2.25	CNTN1
241412_at	-2.04	BTC	204072_s_at	2.24	FRY	219338_s_at	2.25	LRRC49
210563_x_at	-2.04	CFLAR	226666_at	2.24	DAAM1	210657_s_at	2.24	SEP4

225961_at	-2.04	KLHL42	218918_at	2.24	MAN1C1	1558404_at	2.24	LINC00622
226377_at	-2.04	NFIC	231504_at	2.23	CCDC148	202619_s_at	2.24	PLOD2
201502_s_at	-2.04	NFKBIA	229460_at	2.23	FAM126B	224650_at	2.23	MAL2
201331_s_at	-2.04	STAT6	202508_s_at	2.23	SNAP25	204614_at	2.23	SERPINB2
227188_at	-2.06	EVA1C	217414_x_at	2.22	HBA2	1552521_a_at	2.23	TMEM74
58916_at	-2.06	KCTD14	208369_s_at	2.21	GCDH	236407_at	2.22	KCNE1
224895_at	-2.06	YAP1	212774_at	2.20	ZBTB18	220334_at	2.22	RGS17
224894_at	-2.06	YAP1	205259_at	2.19	NR3C2	223869_at	2.22	SOST
235005_at	-2.07	DIS3L	229669_at	2.19	LOC100507263	227909_at	2.21	LINC00087
231899_at	-2.07	ZC3H12C	210971_s_at	2.19	ARNTL	1556499_s_at	2.2	COL1A1
227055_at	-2.08	METTL7B	220761_s_at	2.19	TAOK3	221152_at	2.2	COL8A1
1558292_s_at	-2.08	PIGW	239835_at	2.19	KBTBD8	228101_at	2.18	APBA1
1557014_a_at	-2.09	FAM201A	220047_at	2.17	SIRT4	228695_at	2.18	C8orf46
204780_s_at	-2.09	FAS	205858_at	2.17	NGFR	203913_s_at	2.18	HPGD
218826_at	-2.09	SLC35F2	238682_at	2.17	CCDC96	202149_at	2.18	NEDD9
239579_at	-2.1	EPHX4	209305_s_at	2.17	GADD45B	208607_s_at	2.18	SAA2
232271_at	-2.1	HNF4G	243610_at	2.15	C9orf135	36553_at	2.17	ASMTL
244467_at	-2.1	LOC100510485	213348_at	2.15	CDKN1C	205749_at	2.17	CYP1A1
227174_at	-2.1	WDR72	210657_s_at	2.14	SEP4	1559213_at	2.17	LINC01419
225681_at	-2.11	CTHRC1	221903_s_at	2.13	CYLD	1558678_s_at	2.17	MALAT1
209124_at	-2.11	MYD88	203304_at	2.13	BAMBI	1569020_at	2.17	NEDD9
226895_at	-2.11	NFIC	212912_at	2.13	RPS6KA2	34408_at	2.17	RTN2
206020_at	-2.11	SOCS6	213295_at	2.12	CYLD	1557523_at	2.16	ATP6AP1L
219749_at	-2.12	SH2D4A	219225_at	2.12	PGBD5	201565_s_at	2.16	ID2
229221_at	-2.13	CD44	209160_at	2.11	AKR1C3	213711_at	2.16	KRT81
223895_s_at	-2.13	EPN3	210785_s_at	2.11	THEMIS2	224392_s_at	2.16	OPN3
222664_at	-2.13	KCTD15	222456_s_at	2.10	LIMA1	214247_s_at	2.15	DKK3
221816_s_at	-2.13	PHF11	216060_s_at	2.10	DAAM1	234513_at	2.15	ELOVL3
209679_s_at	-2.13	SMAGP	228645_at	2.10	SNHG9	236289_at	2.15	LOC100506563
225417_at	-2.14	EPC1	205637_s_at	2.10	SH3GL3	236302_at	2.15	PPM1E
226267_at	-2.16	JDP2	221563_at	2.10	DUSP10	203407_at	2.14	PPL
228752_at	-2.17	CRACR2A	200666_s_at	2.09	DNAJB1	201109_s_at	2.14	THBS1
223794_at	-2.18	ARMC4	205352_at	2.08	SERPINI1	53991_at	2.13	DENND2A
232129_s_at	-2.18	LZTS2	238275_at	2.08	HAP1	229976_at	2.13	MORN5
225688_s_at	-2.18	PHLDB2	226208_at	2.07	ZSWIM6	219287_at	2.12	KCNMB4
238043_at	-2.19	ARID1B	201150_s_at	2.07	TIMP3	1552477_a_at	2.11	IRF6
204781_s_at	-2.19	FAS	205569_at	2.06	LAMP3	220957_at	2.1	CTAGE1
1569322_at	-2.19	LINC00857	230951_at	2.06	EPB41L5	231292_at	2.1	EID3
219924_s_at	-2.19	ZMYM6	213300_at	2.06	ATG2A	239533_at	2.1	GPR155
202136_at	-2.19	ZMYND11	226039_at	2.05	MGAT4A	226258_at	2.09	AMN1
203741_s_at	-2.2	ADCY7	210105_s_at	2.05	FYN	219833_s_at	2.08	EFHC1
202417_at	-2.2	KEAP1	209458_x_at	2.05	HBA2	1562921_at	2.08	EP300-AS1
219342_at	-2.21	CASD1	1556129_at	2.05	LOC642533	207980_s_at	2.07	CITED2
202318_s_at	-2.21	SENP6	223129_x_at	2.04	MYLIP	229656_s_at	2.07	EML6
207992_s_at	-2.22	AMPD3	209604_s_at	2.04	GATA3	235944_at	2.07	HMCN1
206033_s_at	-2.22	DSC3	204546_at	2.02	KIAA0513	223822_at	2.07	SUSD4
1552947_x_at	-2.22	ZNF114	211538_s_at	2.02	HSPA2	209277_at	2.07	TFPI2
226129_at	-2.23	FAM83H	220331_at	2.02	CYP46A1	202150_s_at	2.06	NEDD9
219545_at	-2.23	KCTD14	204458_at	2.02	PLA2G15	210195_s_at	2.06	PSG1
205466_s_at	-2.24	HS3ST1	232562_at	2.02	OTUD7A	218764_at	2.05	PRKCH
1552575_a_at	-2.25	C6orf141	225597_at	2.01	SLC45A4	210538_s_at	2.04	BIRC3
213912_at	-2.25	TBC1D30	228062_at	2.01	NAP1L5	230900_at	2.04	CCDC110
223694_at	-2.25	TRIM7	203855_at	2.01	WDR47	236639_at	2.04	LOC100507596
1554014_at	-2.26	CHD2	242814_at	2.00	SERPINB9	36554_at	2.03	ASMTL
226252_at	-2.26	ZBTB20	215321_at	2.00	RUNDC3B	203477_at	2.03	COL15A1
213913_s_at	-2.27	TBC1D30	222701_s_at	2.00	CHCHD7	225373_at	2.02	C10orf54
217523_at	-2.28	CD44	225868_at	-2.06	TRIM47	244407_at	2.02	CYP39A1
227542_at	-2.28	SOCS6	209290_s_at	-2.06	NFIB	236656_s_at	2.02	LOC100288911
213355_at	-2.28	ST3GAL6	226135_at	-2.06	UHRF1BP1	223940_x_at	2.02	MALAT1
210664_s_at	-2.28	TFPI	204779_s_at	-2.06	HOXB7	206197_at	2.02	NME5
226250_at	-2.28	ZBTB20	203453_at	-2.07	SCNN1A	235004_at	2.02	RBM24
226817_at	-2.29	DSC2	223474_at	-2.07	IRF2BPL	206518_s_at	2.02	RGS9
221081_s_at	-2.3	DENND2D	223642_at	-2.07	ZIC2	219487_at	2.01	BBS10

218898_at	-2.3	FAM57A	215022_x_at	-2.07	ZNF33B	226657_at	2.01	NATD1
225996_at	-2.3	LONRF2	212553_at	-2.07	RPRD2	240187_at	2.01	PPP1R3C
231876_at	-2.3	TRIM56	227919_at	-2.08	UCA1	235874_at	2	PRSS35
215022_x_at	-2.31	ZNF33B	1555831_s_at	-2.08	LRRC41	203889_at	2	SCG5
219580_s_at	-2.32	TMC5	232067_at	-2.08	FAXC	235609_at	-2.01	BRIP1
226820_at	-2.32	ZNF362	213113_s_at	-2.08	SLC43A3	204759_at	-2.01	RCBTB2
228999_at	-2.33	CHD2	223282_at	-2.08	TSHZ1	227211_at	-2.02	PHF19
225021_at	-2.33	ZNF532	204420_at	-2.08	FOSL1	203729_at	-2.03	EMP3
1559072_a_at	-2.35	ELFN2	224764_at	-2.08	ARHGAP21	226781_at	-2.04	C7orf55
222016_s_at	-2.35	ZSCAN31	225589_at	-2.08	SH3RF1	229450_at	-2.05	IFIT3
209239_at	-2.36	NFKB1	217853_at	-2.09	TNS3	227873_at	-2.05	TXNDC15
1552946_at	-2.37	ZNF114	203567_s_at	-2.09	TRIM38	220643_s_at	-2.06	FAIM
1552546_a_at	-2.38	LETM2	211518_s_at	-2.09	BMP4	205034_at	-2.07	CCNE2
224473_x_at	-2.38	LZTS2	206848_at	-2.09	COX20	204747_at	-2.08	IFIT3
203453_at	-2.38	SCNN1A	223974_at	-2.10	DLGAP1-AS2	242931_at	-2.08	LONRF3
213153_at	-2.38	SETD1B	211330_s_at	-2.10	HFE	226757_at	-2.09	IFIT2
230748_at	-2.38	SLC16A6	226252_at	-2.10	ZBTB20	238576_at	-2.09	MOCOS
209506_s_at	-2.39	NR2F1	210135_s_at	-2.10	SHOX2	242521_at	-2.17	LOC100505812
203964_at	-2.44	NMI	204011_at	-2.10	SPRY2	218898_at	-2.24	FAM57A
209505_at	-2.45	NR2F1	226820_at	-2.11	ZNF362	227212_s_at	-2.24	PHF19
210942_s_at	-2.45	ST3GAL6	229429_x_at	-2.11	LINC00623	217761_at	-2.36	ADI1
234936_s_at	-2.48	CC2D2A	213939_s_at	-2.11	RUFY3	219352_at	-2.4	HERC6
1557638_at	-2.48	LOC100287676	235046_at	-2.11	INPP4B	202659_at	-2.42	PSMB10
218983_at	-2.49	C1RL	216973_s_at	-2.12	HOXB7	225083_at	-2.48	GTF3C6
227001_at	-2.49	NIPAL2	203275_at	-2.12	IRF2	221816_s_at	-2.49	PHF11
206600_s_at	-2.49	SLC16A5	238513_at	-2.13	PRRG4	242625_at	-2.49	RSAD2
1557137_at	-2.49	TMEM17	239913_at	-2.13	SLC10A4	213797_at	-2.5	RSAD2
220643_s_at	-2.51	FAIM	228365_at	-2.13	CPNE8	235048_at	-2.51	FAM169A
203005_at	-2.51	LTBR	213152_s_at	-2.13	SRSF8	223608_at	-2.57	EFCAB2
238689_at	-2.53	GPR110	238419_at	-2.13	PHLDB2	202589_at	-2.62	TYMS
206858_s_at	-2.53	HOXC6	225091_at	-2.13	ZCCHC3	1554696_s_at	-2.68	TYMS
229022_at	-2.53	ZFX	225512_at	-2.14	ZBTB38	205302_at	-2.69	IGFBP1
225415_at	-2.54	DTX3L	205891_at	-2.14	ADORA2B	225533_at	-2.75	PHF19
235542_at	-2.55	TET3	227049_at	-2.14	ZADH2	203964_at	-2.91	NMI
204401_at	-2.56	KCNN4	220617_s_at	-2.14	ZNF532	219911_s_at	-3.92	SLCO4A1
227506_at	-2.56	SLC16A9	227978_s_at	-2.15	ZADH2			
229450_at	-2.58	IFIT3	232230_at	-2.15	LINC00263			
225381_at	-2.58	MIR100HG	213618_at	-2.15	ARAP2			
205366_s_at	-2.59	HOXB6	234936_s_at	-2.15	CC2D2A			
57715_at	-2.6	CALHM2	225681_at	-2.15	CTHRC1			
204686_at	-2.63	IRS1	218513_at	-2.16	TMA16			
222668_at	-2.63	KCTD15	225457_s_at	-2.17	LINC00263			
238025_at	-2.63	MLKL	241412_at	-2.17	BTC			
213156_at	-2.66	ZBTB20	242005_at	-2.18	LINC00973			
223276_at	-2.67	SMIM3	202606_s_at	-2.18	TLK1			
209373_at	-2.71	MALL	226764_at	-2.18	ZNF827			
202481_at	-2.76	DHRS3	223027_at	-2.19	SNX9			
206155_at	-2.77	ABCC2	210649_s_at	-2.19	ARID1A			
202600_s_at	-2.8	NRIP1	223794_at	-2.19	ARMC4			
221565_s_at	-2.83	CALHM2	209406_at	-2.19	BAG2			
205767_at	-2.84	EREG	238444_at	-2.19	ZNF618			
218963_s_at	-2.84	KRT23	204352_at	-2.19	TRAF5			
209129_at	-2.84	TRIP6	1554020_at	-2.19	BICD1			
213376_at	-2.84	ZBTB1	226592_at	-2.20	ZNF618			
202599_s_at	-2.85	NRIP1	226377_at	-2.20	NFIC			
205266_at	-2.86	LIF	236554_x_at	-2.20	TMC8			
207360_s_at	-2.87	NTSR1	226250_at	-2.20	ZBTB20			
213698_at	-2.87	ZMYM6	204751_x_at	-2.20	DSC2			
223843_at	-2.88	SCARA3	213032_at	-2.21	NFIB			
209431_s_at	-2.89	PATZ1	1552946_at	-2.21	ZNF114			
226284_at	-2.89	ZBTB2	210563_x_at	-2.21	CFLAR			
238029_s_at	-2.97	SLC16A14	230252_at	-2.22	LPAR5			
222062_at	-3	IL27RA	235005_at	-2.22	DIS3L			

235521_at	-3.01	HOXA3	227628_at	-2.22	GPX8			
205479_s_at	-3.01	PLAU	214791_at	-2.23	SP140L			
213258_at	-3.01	TFPI	226143_at	-2.23	RAI1			
209049_s_at	-3.01	ZMYND8	215411_s_at	-2.23	TRAF3IP2			
209048_s_at	-3.01	ZMYND8	224177_s_at	-2.23	PBDC1			
229402_at	-3.02	SAMD13	216942_s_at	-2.23	CD58			
229667_s_at	-3.03	HOXB8	227174_at	-2.23	WDR72			
210273_at	-3.07	PCDH7	223591_at	-2.24	RNF135			
212660_at	-3.08	JADE2	235109_at	-2.24	ZBED3			
206785_s_at	-3.08	KLRC2	227607_at	-2.24	STAMBPL1			
227954_at	-3.15	ITPRIPL2	223595_at	-2.25	TMEM133			
211302_s_at	-3.17	PDE4B	224579_at	-2.25	SLC38A1			
241902_at	-3.18	MKX	207614_s_at	-2.25	CUL1			
232202_at	-3.22	FAM83B	227294_at	-2.25	ZNF689			
242979_at	-3.34	IRS1	1557014_a_at	-2.26	FAM201A			
206504_at	-3.58	CYP24A1	207723_s_at	-2.26	KLRC3			
1568619_s_at	-3.81	ITPRIPL2	59625_at	-2.26	NOL3			
227514_at	-3.88	ITPRIPL2	1569322_at	-2.26	LINC00857			
227792_at	-3.99	ITPRIPL2	1568609_s_at	-2.26	LINC00623			
225645_at	-4.09	EHF	228046_at	-2.26	ZNF827			
228640_at	-4.24	PCDH7	243618_s_at	-2.27	ZNF827			
219836_at	-4.24	ZBED2	231864_at	-2.27	ZNF33A			
239468_at	-4.57	MKX	238520_at	-2.27	TRERF1			
208025_s_at	-5.02	HMGA2	231851_at	-2.27	RAVER2			
203708_at	-5.78	PDE4B	232752_at	-2.28	LOXL1-AS1			
			219749_at	-2.28	SH2D4A			
			243299_at	-2.28	VRK2			
			1557137_at	-2.28	TMEM17			
			235124_at	-2.28	EIF3J-AS1			
			226895_at	-2.29	NFIC			
			212762_s_at	-2.29	TCF7L2			
			212606_at	-2.29	WDFY3			
			207029_at	-2.30	KITLG			
			209921_at	-2.31	SLC7A11			
			202241_at	-2.31	TRIB1			
			229221_at	-2.32	CD44			
			206032_at	-2.32	DSC3			
			223895_s_at	-2.32	EPN3			
			226555_at	-2.33	INO80D			
			218826_at	-2.33	SLC35F2			
			225996_at	-2.33	LONRF2			
			223761_at	-2.33	FGF19			
			238025_at	-2.34	MLKL			
			233329_s_at	-2.34	KRCC1			
			224895_at	-2.34	YAP1			
			210664_s_at	-2.35	TFPI			
			210845_s_at	-2.35	PLAUR			
			224765_at	-2.35	MSL1			
			212096_s_at	-2.35	MTUS1			
			202863_at	-2.36	SP100			
			218284_at	-2.36	SMAD3			
			204750_s_at	-2.36	DSC2			
			1553960_at	-2.36	SNX21			
			209124_at	-2.37	MYD88			
			227055_at	-2.37	METTL7B			
			55081_at	-2.38	MICALL1			
			213342_at	-2.38	YAP1			
			220030_at	-2.38	STYK1			
			202319_at	-2.38	SENP6			
			234969_s_at	-2.38	EPC1			
			213150_at	-2.39	HOXA10			
			1555881_s_at	-2.39	LZTS2			
			226129_at	-2.39	FAM83H			

			221935_s_at	-2.39	EOGT			
			217678_at	-2.40	SLC7A11			
			219342_at	-2.40	CASD1			
			204780_s_at	-2.41	FAS			
			209905_at	-2.41	HOXA10			
			235048_at	-2.42	FAM169A			
			211744_s_at	-2.43	CD58			
			225732_at	-2.43	KLHL42			
			228494_at	-2.44	PPP1R9A			
			225961_at	-2.44	KLHL42			
			205173_x_at	-2.44	CD58			
			209679_s_at	-2.44	SMAGP			
			223592_s_at	-2.44	RNF135			
			1558292_s_at	-2.45	PIGW			
			209939_x_at	-2.45	CFLAR			
			235199_at	-2.46	RNF125			
			227474_at	-2.47	PAX8-AS1			
			227624_at	-2.47	TET2			
			216493_s_at	-2.47	IGF2BP3			
			202417_at	-2.48	KEAP1			
			228752_at	-2.48	CRACR2A			
			1557638_at	-2.48	LOC100287676			
			215073_s_at	-2.48	NR2F2			
			219924_s_at	-2.49	ZMYM6			
			223294_at	-2.49	PBDC1			
			221081_s_at	-2.50	DENND2D			
			227188_at	-2.51	EVA1C			
			219543_at	-2.51	PBLD			
			225021_at	-2.52	ZNF532			
			224473_x_at	-2.52	LZTS2			
			223541_at	-2.52	HAS3			
			228899_at	-2.52	LOC100132884			
			203780_at	-2.53	MPZL2			
			226817_at	-2.54	DSC2			
			224022_x_at	-2.54	WNT16			
			208944_at	-2.54	TGFBR2			
			230748_at	-2.54	SLC16A6			
			215719_x_at	-2.55	FAS			
			228141_at	-2.55	GPX8			
			228304_at	-2.56	RBM43			
			227578_at	-2.56	TMPO-AS1			
			221865_at	-2.58	C9orf91			
			206155_at	-2.58	ABCC2			
			239579_at	-2.59	EPHX4			
			218898_at	-2.61	FAM57A			
			213844_at	-2.61	HOXA5			
			206600_s_at	-2.61	SLC16A5			
			236207_at	-2.62	SSFA2			
			201324_at	-2.63	EMP1			
			224894_at	-2.63	YAP1			
			209239_at	-2.63	NFKB1			
			219580_s_at	-2.64	TMC5			
			207992_s_at	-2.64	AMPD3			
			204401_at	-2.65	KCNN4			
			209373_at	-2.66	MALL			
			203964_at	-2.67	NMI			
			203819_s_at	-2.67	IGF2BP3			
			226534_at	-2.68	KITLG			
			201170_s_at	-2.68	BHLHE40			
			225688_s_at	-2.69	PHLDB2			
			212867_at	-2.69	NCOA2			
			204781_s_at	-2.69	FAS			
			226413_at	-2.69	LINC00938			

			218917_s_at	-2.70	ARID1A			
			204619_s_at	-2.71	VCAN			
			238689_at	-2.71	GPR110			
			227445_at	-2.72	ZNF689			
			223843_at	-2.74	SCARA3			
			238043_at	-2.75	ARID1B			
			1554014_at	-2.76	CHD2			
			1553172_at	-2.76	ZNF777			
			231876_at	-2.76	TRIM56			
			203741_s_at	-2.77	ADCY7			
			222062_at	-2.77	IL27RA			
			225417_at	-2.78	EPC1			
			218704_at	-2.79	RNF43			
			203005_at	-2.80	LTBR			
			214678_x_at	-2.81	ZFX			
			209506_s_at	-2.81	NR2F1			
			1559072_a_at	-2.81	ELFN2			
			229450_at	-2.82	IFIT3			
			209129_at	-2.84	TRIP6			
			205266_at	-2.84	LIF			
			213913_s_at	-2.88	TBC1D30			
			226267_at	-2.88	JDP2			
			206858_s_at	-2.89	HOXC6			
			228280_at	-2.90	ZC3HAV1L			
			213156_at	-2.91	ZBTB20			
			222668_at	-2.91	KCTD15			
			213698_at	-2.91	ZMYM6			
			1552546_a_at	-2.92	LETM2			
			217523_at	-2.92	CD44			
			228999_at	-2.94	CHD2			
			226284_at	-2.94	ZBTB2			
			213912_at	-2.94	TBC1D30			
			217999_s_at	-2.95	PHLDA1			
			220643_s_at	-2.97	FAIM			
			207360_s_at	-2.97	NTSR1			
			218963_s_at	-2.97	KRT23			
			210993_s_at	-3.00	SMAD1			
			57715_at	-3.01	CALHM2			
			210942_s_at	-3.02	ST3GAL6			
			226490_at	-3.02	NHSL1			
			213153_at	-3.05	SETD1B			
			211124_s_at	-3.06	KITLG			
			202318_s_at	-3.07	SENP6			
			1552575_a_at	-3.07	C6orf141			
			218983_at	-3.07	C1RL			
			213355_at	-3.08	ST3GAL6			
			225381_at	-3.08	MIR100HG			
			225415_at	-3.09	DTX3L			
			205366_s_at	-3.10	HOXB6			
			227506_at	-3.10	SLC16A9			
			202481_at	-3.11	DHRS3			
			219545_at	-3.13	KCTD14			
			1566901_at	-3.14	TGIF1			
			223276_at	-3.17	SMIM3			
			210273_at	-3.18	PCDH7			
			209431_s_at	-3.19	PATZ1			
			205479_s_at	-3.19	PLAU			
			222016_s_at	-3.19	ZSCAN31			
			204686_at	-3.21	IRS1			
			213258_at	-3.22	TFPI			
			217996_at	-3.28	PHLDA1			
			225181_at	-3.28	ARID1B			
			227798_at	-3.31	SMAD1			

			205466_s_at	-3.34	HS3ST1			
			227954_at	-3.34	ITPRIPL2			
			203820_s_at	-3.38	IGF2BP3			
			206020_at	-3.39	SOCS6			
			235521_at	-3.41	HOXA3			
			221565_s_at	-3.42	CALHM2			
			213376_at	-3.44	ZBTB1			
			235542_at	-3.44	TET3			
			58916_at	-3.44	KCTD14			
			231899_at	-3.50	ZC3H12C			
			209505_at	-3.51	NR2F1			
			229022_at	-3.52	ZFX			
			206785_s_at	-3.56	KLRC2			
			212660_at	-3.58	JADE2			
			241902_at	-3.62	MKX			
			209048_s_at	-3.66	ZMYND8			
			227542_at	-3.66	SOCS6			
			238029_s_at	-3.74	SLC16A14			
			242979_at	-3.77	IRS1			
			217997_at	-3.79	PHLDA1			
			229402_at	-3.83	SAMD13			
			211302_s_at	-3.87	PDE4B			
			202600_s_at	-3.92	NRIP1			
			219836_at	-3.94	ZBED2			
			203313_s_at	-3.97	TGIF1			
			209049_s_at	-4.00	ZMYND8			
			1568619_s_at	-4.00	ITPRIPL2			
			229667_s_at	-4.10	HOXB8			
			227514_at	-4.17	ITPRIPL2			
			205767_at	-4.17	EREG			
			232202_at	-4.18	FAM83B			
			227792_at	-4.43	ITPRIPL2			
			225645_at	-4.74	EHF			
			202599_s_at	-4.82	NRIP1			
			206504_at	-4.86	CYP24A1			
			239468_at	-5.04	MKX			
			228640_at	-5.65	PCDH7			
			208025_s_at	-5.96	HMGA2			
			203708_at	-6.74	PDE4B			

Table S2 The interaction profile of TOP2B and drugs by iGEMDOCK analysis

Drug	Total fitness energy	VDW	Hbond	Elect
Etoposide	-116.23	-89.14	-27.09	0
Mitomycin C	-92.88	-61.62	-31.61	0

Note: Fitness (kcal/mol) is the total energy of a predicted pose in the binding site and it is calculated by the following equation: Fitness = vdW + Hbond + Elect. The terms, vdW, Hbond, and Elect, indicate van der Waal energy, hydrogen bonding energy, and Elect term is electro statistic energy, respectively
